# New Perspectives on Predicting and Modifying Cognition in Older Adulthood

**DOI:** 10.1093/geroni/igaa057.3202

**Published:** 2020-12-16

**Authors:** Allison Bielak

**Affiliations:** Colorado State University, Fort Collins, Colorado, United States

## Abstract

The underlying theme of my research is to advance our understanding of cognitive aging through early detection of cognitive decline and the promotion of positive lifestyle factors. I will present my past and recent work that uses new perspectives to predict and modify cognition with older age, specifically focusing on intraindividual variability in cognitive speed, and lifestyle activity engagement. This includes examining the utility of variability in cognitive speed, usually a predictor of decline, instead as a predictor of cognitive plasticity following activity interventions. Further, the association between activity engagement and later cognitive ability and dementia risk is more complex than first appears. I will present research that looks at critical issues about the activity-cognition relation from a different perspective, including unique frameworks investigating the ideal way to measure activity, and the duration of how long past activity can potentially impact cognition. Finally, I will discuss my future research directions.

